# P-772. Whole Genome Analysis of Mycobacterium intracellulare isolates from Colonic Biopsy In Inflammatory Bowel Disease Patients

**DOI:** 10.1093/ofid/ofae631.967

**Published:** 2025-01-29

**Authors:** Urvashi B Singh, Sushanta Deb, Lata Kumari, Neeraj Mahajan, Vineet Ahuja

**Affiliations:** All India Institute of Medical Sciences ,New Delhi, New Delhi, Delhi, India; Washington State University, Pullman, Washington; All India Institute of Medical Sciences,New Delhi, New Delhi, Delhi, India; All India Institute of Medical Sciences, New Delhi, New Delhi, Delhi, India; ALL INDIA INSTITUTE OF MEDICAL SCIENCES, NEW DELHI, DELHI, Delhi, India

## Abstract

**Background:**

A long debate has prevailed over cause of Crohn's disease with published evidence for Mycobacterium paratuberculosis (MAP).Our study was designed to look for mycobacterial pathogens by culture and DNA PCR in patients with inflammatory bowel disease(IBD).

Phylogenetic Analysis of M. intracellulare isolate genomes compared with reference M. intracellular strain ATCC 13950T

**Figure:**
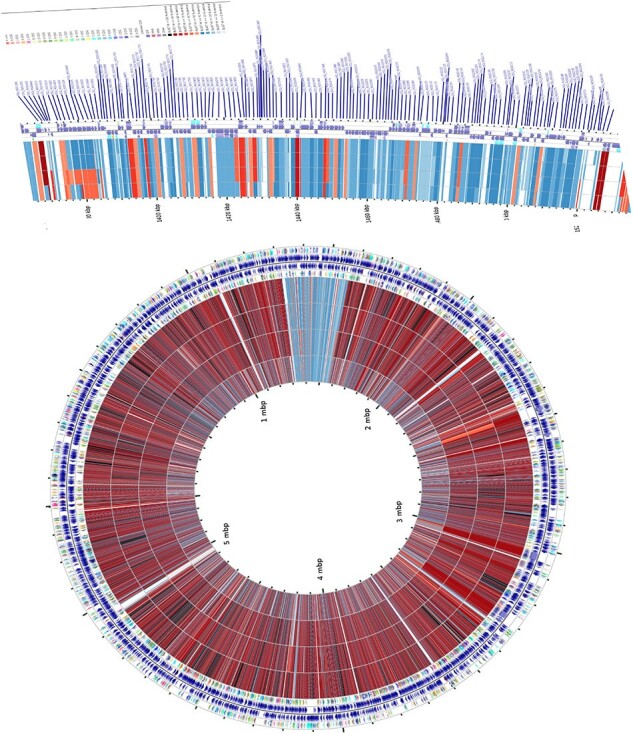

Circular representation of four M. intracellulare isolate genomes compared with reference M. intracellular strain ATCC 13950T. Inner to outer four concentric rings show isolates from this study. The information is read from outer circle to inner as follows: genome size, genes on the forward strand, genes on the reverse strand, t-RNA, and r-RNA of reference strain.The percentage similarity is represented by different color codes. Zoomed portion represent absent genetic region in four isolates.

**Methods:**

Colonic biopsy and Blood Buffy coat samples were processed for cultures and DNA detection for mycobacterial pathogens (including MAP) in 889 patients with inflammatory bowel disease; Crohn’s disease (CD)( clinical manifestations, endoscopic,radiological and histological features as per ECCO consensus guidelines), Intestinal tuberculosis,Controls (Adult patients with hemorrhoidal bleeding and no other colonic disease undergoing sigmoidoscopy).

**Results:**

Four cultures from CD patients grew Mycobacterium intracellulare. Whole Genome Sequencing identified deletion regions in the studied isolates, with 156 protein-coding genes absent in all four isolates compared to the reference genome (M. intracellulare Strain ATCC 13950T). Antibiotic resistance and virulence factor analyses indicated resistance to rifampicin and isoniazid and an abundance of virulence factor genes in M. Intracellulare species. Pan and core gene analyses suggested a closed pan-genome in M.intracellulare, with limited distribution of antibiotic resistance genes.

**Conclusion:**

M. Intracellulare is reported in immune-compromised patientsin several studies. The results of the core genome phylogenetic analysis suggest that factors beyond the isolation sources of M.intracellulare strains may contribute to shape their evolutionary trajectory. Functional analysis indicated a gradual decrease in metabolic capacity across clusters, suggesting an ongoing genome streamlining process in M. Intracellulare species. Essential gene prediction identified strain specific essential genes, emphasizing the importance of energy-related processes. Homologous recombination rate analysis suggested recombination in M. intracellulare strains, with specific parameters showing variation compared to other pathogenic species. This comprehensive study contributes valuable information about the genomic characteristics, evolution, and functional profiles of M. intracellulare strains.

**Disclosures:**

**All Authors**: No reported disclosures

